# KEAP1 overexpression is correlated with poor prognosis and immune infiltration in liver hepatocellular carcinoma

**DOI:** 10.3389/fmed.2024.1391843

**Published:** 2024-06-13

**Authors:** Xin Wei, Yigui Tang, Meijuan Zheng, Yuanhong Xu, Zhongxin Wang

**Affiliations:** Department of Clinical Laboratory, The First Affiliated Hospital of Anhui Medical University, Hefei, China

**Keywords:** KEAP1, LIHC, biomarker, immune infiltration, liver function

## Abstract

**Purpose:**

Liver hepatocellular carcinoma (LIHC) is the most common type of liver cancer, but there is a lack of effective indicators for its early diagnosis and prognosis, so we explored the role of KEAP1 in LIHC patients in this study.

**Methods:**

The Cancer Genome Atlas (TCGA) dataset was used to investigate the relationship between KEAP1 expression and clinicopathological features and prognosis of LIHC patients. KEAP1 expression related pathways were enriched by Gene Ontology (GO) and gene set enrichment analysis (GSEA). Besides, KEAP1 expression-related immune infiltration was performed by single-sample GSEA (ssGSEA), and function of immune cells was detected by flow cytometry.

**Results:**

It was found that KEAP1 expression was significantly increased and correlated with overall survival of LIHC patients. A total of 231 differentially expressed genes (DEGs) between LIHC patients with high- and low-KEAP1 expression were found, which associated with various biological pathways. Besides, KEAP1 expression was positively correlated with the infiltration level of T helper cells and Th2 cells but negatively correlated with DCs and cytotoxic cells. Functional analysis revealed that the expression of IL 4 in Th2 cells and CD107a, GrA and GrB in cytotoxic cells was significantly greater in LIHC patients than in HCs. In addition, KEAP1 expression was closely correlated with liver function in LIHC patients.

**Conclusion:**

Highly expressed KEAP1 was closely related to the diagnosis, prognosis, immune cell infiltration, and liver function of LIHC, which might promote the progression of LIHC through regulating cell development, signal transduction, and abnormal immune response. The current study partially revealed the role of KEAP1 in LIHC and provided a potential biomarker for the diagnosis, prognosis and treatment of LIHC.

## 1 Introduction

Liver cancer is the sixth most common malignancy and the fourth leading cause of malignancy-related death worldwide, with 841,080 new cases in 2018. It is estimated that more than 1 million people will develop liver cancer each year by 2025. Liver cancer remains a global challenge, and its incidence is growing worldwide. Liver hepatocellular carcinoma (LIHC) is the most common type of liver cancer, accounting for approximately 90% of the total cases. Despite the growing need for molecular identification of tumors using tissue biopsies in clinical practice, LIHC diagnosis is often based on noninvasive criteria. Potentially actionable mutations are present in approximately 25% of LIHC; however, this has not yet been translated into clinical practice because molecular information from tissue or liquid biopsies is needed, so noninvasive diagnosis remains a challenge at present (1–3). In addition, effective indicators for the early diagnosis, prognosis and treatment of LIHC are lacking, so it is urgent to explore new molecular targets.

Kelch-like ECH-associated Protein 1 (KEAP1), a Cullin 3-based E3 ubiquitin ligase adaptor subunit, senses a variety of abnormal signals, including oxidation and electrophilic stimulation. The main function of KEAP1 is to regulate the activity of NF-E2-related molecule 2 (Nrf2) and affect the expression of its downstream molecules so as to play different immunomodulatory roles (4). It has been reported that KEAP1 plays an important regulatory role in the occurrence and development of many liver diseases. After competitively binding to KEAP1, P62 can promote an increase of Nrf2 expression, activate the expression of its downstream related molecules (including NQO1, HO1 and FTH1, etc.), inhibit the apoptosis of liver cancer cells induced by iron accumulation, and may participate in promoting the continuous progression of liver cancer (5). Expression of KEAP1 was significantly increased in patients with primary biliary cirrhosis, while the expression of Nrf2 was significantly decreased, resulting in decreased expression of its downstream related molecules (HO-1 and GCLC), which may inhibit oxidative stress in the liver and promote the progression of cirrhosis (6). Our study also revealed that KEAP1 and related target genes were abnormally expressed in hepatocellular carcinoma cells and were closely related to drug resistance in hepatocellular carcinoma (7). However, the association between KEAP1 and LIHC has not yet been characterized.

In this study, we sought to demonstrate the association between KEAP1 and LIHC, and analyzed the prognostic role of KEAP1 in LIHC based on RNA-sequencing (RNA-seq) data from The Cancer Genome Atlas (TCGA). We first analyzed the changes of KEAP1 expression in LIHC, and analyzed the correlation between KEAP1 expression and the severity and prognosis of LIHC. Then, we screened the DEGs associated with KEAP1 expression in LIHC, and performed Gene Ontology (GO) and gene set enrichment analysis (GSEA) on these genes. In addition, we analyzed the relationship between KEAP1 expression and immune infiltration, and further examined the functional changes in related immune cells in LIHC patients. Finally, we analyzed the association between KEAP1 expression and liver function in LIHC patients. This study may provide novel insight into the underlying mechanisms of LIHC tumorigenesis and may have positive implications for enriching the diagnosis, prognosis and treatment of LIHC.

## 2 Materials and methods

### 2.1 Data acquisition

Datasets from the TCGA database^1^ were included: gene expression data (HTSeq-Counts and HTSeq-FPKM [high-throughput sequencing fragments per kilobase per million]) and the corresponding detailed clinical data from LIHC samples. Level 3 HTSeq-FPKM data were transformed into transcripts per million reads (TPM). 374 LIHC cases and 50 normal cases were included in our study, cases with insufficient or missing data were removed from subsequent data processing. LIHC patients were classified into low- and high-KEAP1 expression groups according to the median KEAP1 expression value.

### 2.2 Survival analysis

Survival curves were calculated according to the Kaplan–Meier method utilizing the Kaplan–Meier plotter and log-rank test by R (survival) package (V3.6.3)^2^. Cox proportional hazards models estimating the hazard ratio (HR) were established to determine whether KEAP1 was associated with the survival events. HR with 95% confidence intervals and log-rank *p*-value were calculated via univariate survival analysis (8). The nomogram combining the expression of KEAP1 and clinicopathological risk factors was constructed with the “rms” package and quantitatively assessed by the concordance index (C-index).

### 2.3 Immune infiltration analysis

The immune infiltration analysis of LIHC for 24 types of immune cells was performed by single-sample gene set enrichment analysis (ssGSEA) method from the GSVA package in R (version 3.6) (9). Markers of the 24 types of immune cells were extracted from the research of Bindea and colleagues (10). Lollipop chart was plotted to examine the correlation of KEAP1 expression with 24 types of immune cell infiltration in LIHC samples with the Spearman correlation analysis. Vioplot was plotted to assess the relationship of KEAP1 and immune cell recruitment with the Wilcoxon rank-sum test.

### 2.4 Gene set enrichment analysis

GSEA is a computational method that determines whether a defined set of genes exhibits statistically significant and concordant differences between two biological states. GSEA^3^ was performed with the R package cluster Profiler (version 3.14.3) to demonstrate the significant functions and pathways between groups expressing high- and low-levels of KEAP1 in LIHC. “c2.cp.v7.2.symbols.gmt [Curated]” from MSigDB Collections^4^ was selected as the reference gene set, false discovery rate (FDR) <0.25 and adjusted *p*-value (p.adj) <0.05 are considered to be significantly enriched (11, 12).

### 2.5 ROS detection

Dulbecco’s Modified Eagle Medium (DMEM, 11966025, Gibco, Grand Island, NY) medium supplemented with 10% FBS (10,099,158, Gibco, Grand Island, NY) was used to adjust the cell concentration at the logarithmic growth stage to 4 × 10^5^/mL. 100 μL of cell suspension was placed in a 96-well round bottom culture plate and cultured at 37°C in a humidified CO_2_ incubator. After 12 h, the experimental group was replaced with DMEM medium supplemented with 10% FBS + 10 mmol/L CCl_4_ (C032225, Koether, Shanghai, China), and the control group was replaced with DMEM medium supplemented with 10% FBS, and 8 parallel wells were made in each group. After 24 h of continuous culture, the cells and culture medium were collected and put into an ultrasonic grinder to crush the cells, and the cell debris were centrifuged at 10000 rpm/min for 10 min, and the supernuant of each group were detected immediately with ROS ELISA kit (EIA06562h, Xinqidi, Wuhan, China), and the experimental operation was carried out in strict accordance with the reagent instructions. Briefly, 100 μL of specimens or ROS standards with different concentrations were added into the corresponding experimental wells, and incubated at 37°C for 90 min; after washing the plate twice with the washing solution, 100 μL of biotinized ROS antibody working solution was added to each well and incubated at 37°C for 60 min; after washing plate with washing solution for three times, 100 μL of enzyme conjugate working solution was added to each well and incubated at 37°C for 30 min without light; after washing plate with washing liquid for 5 times, add 100 μL of TMB color developing working liquid to each well, and incubate at 37°C for 20 min without light; add 100 μL of the termination solution to each well and mix it well, immediately measure ROS level with an microplate reader (Yantai Addcare Bio-tech Co., Ltd. Shandong, China).

### 2.6 Patients

Twenty LIHC patients without autoimmune disease were enrolled at the First Affiliated Hospital of Anhui Medical University. All patients were diagnosed and grouped according to the criteria from Bureau of Medical Administration, National Health Commission of the People’s Republic of China (13). Twenty age- and sex-matched healthy individuals were recruited as healthy controls (HCs). The study was approved by the ethics committee of the First Affiliated Hospital of Anhui Medical University. Whole blood specimens were obtained from all subjects and peripheral blood mononuclear cells (PBMCs) were isolated by Ficoll density gradient centrifugation on Human Lymphocyte Separation Medium (7,111,012, Dakewe, Shenzhen, China). The clinical and laboratory characteristics of the populations enrolled are summarized in Table 1. Detailed data are presented in Supplementary Table S1.

**Table 1:** Clinical characteristics of the populations enrolled in the study.

Group	HC	LIHC
case	20	20
Sex (male/female)	14/6	16/4
Age (years)	55.00 (41–75)	59.50 (45–73)
TP (g/L)	70.15 (50.50–80.60)	66.85 (48.00–78.20)
ALB (g/L)	43.65 (34.30–50.60)	41.05 (30.30–49.00)
TBIL (μmol/L)	12.13 (7.16–36.80)	20.43 (9.36–216.20)
DBIL (μmol/L)	2.26 (1.10–19.90)	4.53 (1.90–179.20)
IBIL (μmol/L)	9.16 (5.14–14.68)	15.28 (7.09–36.98)
ALT (U/L)	20.50 (5.80–36.80)	38.50 (12.00–435.00)
AST (U/L)	12.40 (4.20–20.60)	54.00 (18.00–443.00)
PLT (×10^9^/L)	165.50 (135.00–350.00)	132.50 (41.00–322.00)
PT (s)	10.00 (9.00–13.00)	10.95 (9.90–15.00)
FIB (g/L)	3.50 (1.80–4.00)	2.60 (1.53–4.10)
AFP (mg/L)	3.40 (1.30–8.00)	12.66 (1.30–100000.00)
HBV-DNA (IU/mL)	ND	750 (ND-5 × 10^8^)
HBsAg+	0	20
HBsAb+	20	0
HBeAg+	0	14
HBeAb+	0	6
HBcAb+	0	20

Data are shown as median and range. TP, total protein; ALB, albumin; TBIL, total bilirubin; DBIL, direct bilirubin; IDBL, indirect bilirubin; ALT, alamine aminotransferase; AST, aspartate aminotransferase; PLT, platelet; PT, prothrombin time; FIB, fibrinogen; AFP, alpha fetal protein; ND, not detected; HC, healthy control; LIHC, liver hepatocellular carcinoma.

### 2.7 Western blot

Appropriate amount of liver cancer tissues and adjacent tissues were taken, fully ground on ice and cleaved by RIPA (P0013J, Beyotime, Shanghai, China). After centrifugation, supernatant was taken and the protein concentration was detected by BCA protein quantitative kit (P0010, Beyotime, Shanghai, China). 25 μg of protein was taken for sodium dodecyl sulphate-polyacrylamide gel electrophoresis (SDS-PAGE, P0012A, Beyotime, Shanghai, China), and transferred to nitrocellulosa membrane (FFP26, Beyotime, Shanghai, China), which was closed with western blocking solution (P0023B-500 mL, Beyotime, Shanghai, China) at room temperature for 1 h, and then primary antibodies (KEAP1, 1:1000, WL03285; β-actin, 1:1000, WL01372. Wan Class, Shenyang, China) were added and incubated at 4°C overnight. After western wash buffer (P0023C-1 L, Beyotime, Shanghai, China) washing, the secondary antibodies (1,7,000, WLA023, Wan Class, Shenyang, China) were added and incubated at room temperature for 2 h. After western wash buffer washing, ECL luminescent agent (P0018S, Beyotime, Shanghai, China) was added for exposure, and the optical density (OD) value of protein was analyzed by Image J software (National Institutes of Health, Maryland, United States). β-actin was used as standardized control, the ratio of OD value of KEAP1 to β-actin was used as the relative expression of target protein.

### 2.8 RNA extraction and semiquantitative real-time polymerase chain reaction

Total RNA from PBMCs was isolated using a SteadyPure Tissue and Cell Small RNA Extraction Kit (AG21024, Accurate Biology, Hunan, China) according to the manufacturer’s protocol. Gene expression was evaluated using PrimeScript^™^ RT Master Mix (RR036A, Takara, Japan) and TB Green^™^ Premix Ex Taq^™^ II (RR820A, Takara, Japan). The thermocycling conditions consisted of an initial step of 2 min at 50°C and denaturation for 30 s at 95°C, followed by 40 cycles at 95°C for 5 s and 60°C for 35 s, and a melt curve was generated to confirm the specificity of the PCR products. The KEAP1 sequences used were: sence primer (5′ → 3′), GTGTCCATTGAGGGTATCCACC, and antisence primer (5′ → 3′), GCTCAGCGAAGTTGGCGAT. β-actin was used for normalization, and the sequences were: sence primer (5′ → 3′), AGCCTCGCCTTTGCCGATCCG, and antisence primer (5′ → 3′), TCTCTTGCTCTGGGCCTCGTCG. Each sample was analyzed in triplicate, relative gene expression was analyzed with the comparative Ct method (2^−ΔΔCt^).

### 2.9 Fluorescence-activated cell sorting analysis

Most antibodies were purchased from BioLegend (San Diego, CA), including allophycocyanin (APC)-conjugated anti-human CD45 mAb (368512), fluorescein isothiocyanate (FITC)-conjugated anti-human CD3 mAb (300306), phycoerythrin-Cy7 (PE/Cy7)-conjugated anti-human CD4 mAb (317414), Brilliant Violet 421^™^ (BV-421)-conjugated anti-human CD8 mAb (344748), allophycocyanin-Cy7 (APC/Cy7)-conjugated anti-human CD107a mAb (328630), PE-conjugated anti-human perforin mAb (308106), and peridin chlorophyll protein-Cy5.5 (PerCP/Cy5.5)-conjugated anti-human Granzyme B (GrB) mAb (372212). Besides, PE/Cy7-conjugated anti-human Granzyme A (GrA) mAb from Thermo Fisher Scientific (25-9,117-42, Waltham, MA) and PE-conjugated anti-human IL-4 mAb from BD Pharmingen (559,333, San Jose, CA) were used. We detected the functional properties of cytotoxic T cells as follows: APC/Cy7-conjugated anti-human CD107a mAb, PE-conjugated anti-human perforin mAb, PE/Cy7-conjugated anti-human Granzyme A (GrA) mAb and PerCP/Cy5.5-conjugated anti-human Granzyme B (GrB) mAb. The expression of IL-4 was detected to assist in understanding the function of Th2 cells. PBMCs from all subjects were isolated by Ficoll density gradient centrifugation on Lymphoprep (AS1114546, Axis-shield, Norway). For cell surface staining, 2 × 10^5^ PBMCs were taken into a flow tube, washed with 2 mL of 1× cell staining buffer (420,201, BioLegend, San Diego, CA), centrifuged at 1500 rpm/min for 5 min, and the pellet were resuspended in residual buffer. Add appropriate amount of fluorescent antibodies (FITC-conjugated anti-human CD3 mAb, 5 μL; APC-conjugated anti-human CD45 mAb, 4 μL; PE/Cy7-conjugated anti-human CD4 mAb, 4 μL; BV-421-conjugated anti-human CD8 mAb, 3 μL) and incubated at room temperature for 20 min in the dark. Wash with 2 mL of PBS by centrifugation at 1500 rpm/min for 5 min, fully discard the supernatant, add 200 μL of PBS to resuspend pellet and analyzed with BD FACSCanto II (Becton Dickinson, San Jose, CA) and FlowJo Software (TreeStar, Ashland, OR).

### 2.10 Cell stimulation for intracellular staining

PBMCs (5 × 10^5^) were suspended in 100 μL of Roswell Park Memorial Institute (RPMI)-1640 medium (12,633,012, Gibco, Grand Island, NY) and placed in a 96-well round bottom plate with stimulants (phorbol 12-myristate 13-acetate [PMA, 50 ng/mL] and ionomycin [1 μg/mL]) and GolgiStop (Brefeldin A [5 μg/mL]) (423,303, BioLegend, San Diego, CA) at 37°C in a humidified CO_2_ incubator for 4 h. Then, the PBMCs were harvested and washed twice with 2 mL of 1× cell staining buffer by centrifugation at 1500 rpm/min for 5 min, and the pellet were resuspended in residual buffer. Cell surface staining antibodies were added and incubated at room temperature for 20 min in the dark. Wash twice with 1× cell staining buffer and fully discard the supernatant, add 300 μL of Fixation/Permeabilization solution (554,714, BD Pharmingen, San Jose, CA) and incubated at room temperature for 20 min in the dark. Wash twice with 2 mL of 1× Wash Buffer by centrifugation at 1500 rpm/min for 5 min, and the pellet were resuspended in residual buffer. Add appropriate amount of cytokine antibodies (APC/Cy7-conjugated anti-human CD107a mAb, 4 μL; PE-conjugated anti-human perforin mAb, 3 μL; PerCP/Cy5.5-conjugated anti-human GrB mAb, 4 μL; PE/Cy7-conjugated anti-human GrA mAb, 4 μL; PE-conjugated anti-human IL-4 mAb, 3 μL) and incubated at room temperature for 20 min in the dark. After which, protocols for pellet washing and suspension were carried out as described above and analyzed with BD FACSCanto II and FlowJo Software.

### 2.11 Statistical analysis

Data processing and statistical analysis were performed using R software (version 3.6, R Foundation for Statistical Computing, Vienna, Austria) and GraphPad Prism software (v. 7.0a; GraphPad Software, La Jolla, CA). The Wilcoxon rank-sum test and Wilcoxon signed-rank test were used to analyze the expression of KEAP1 in non-paired and paired samples, respectively. The chi-square test or Fisher’s exact test was used to analyze the correlation between the KEAP1 expression level and clinicopathological parameters of LIHC patients. The Kaplan–Meier method and Cox regression model were used to perform survival analyses. A two-sided *p*-value <0.05 was considered to be significant except where noted. Statistical significance is indicated as follows: ***p < 0.001, **p < 0.01, and *p < 0.05.

## 3 Results

### 3.1 KEAP1 expression was abnormally elevated in LIHC

**Figure 1:**
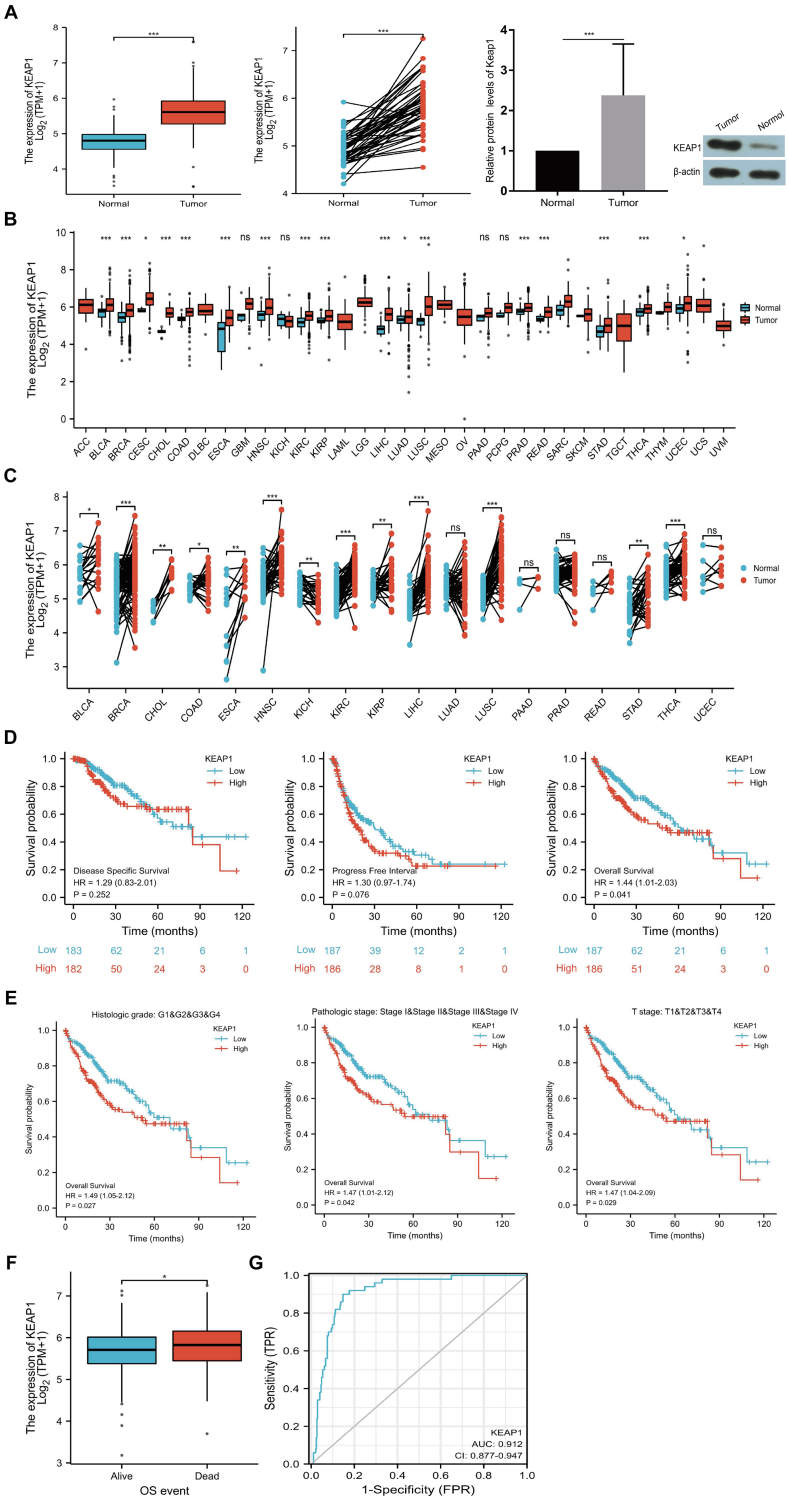
KEAP1 overexpression is closely related to LIHC. **(A)** KEAP1 expression in LIHC patients was significantly increased in both paired and non-paired samples. **(B,C)** KEAP1 expression was elevated in most cancers in both paired and non-paired samples. **(D)** KEAP1 expression is associated with overall survival of LIHC patients. **(E)** KEAP1 expression was correlated with the survival time of LIHC patients with different stages. **(F)** LIHC patients with dead outcome had higher KEAP1 expression than those with alive outcome. **(G)** KEAP1 has good diagnostic value for LIHC. Statistical significance is indicated as follows: ***p < 0.001, **p < 0.01, and *p < 0.05. TPM, transcripts per million reads; HR, hazard ratio; LIHC, liver hepatocellular carcinoma.

KEAP1 expression was significantly higher in LIHC tissues compared with the normal tissues in both non-paired samples (371:160) and paired samples (50:50) based on TCGA databases. Then, we detected KEAP1 expression by western blotting to verify the above results, and found that the expression level of KEAP1 in LIHC tissues was significantly higher than that in adjacent tissues, which was consistent with the above conclusion (Figure 1A). In addition, we analyzed KEAP1 expression in pancarcinoma, and found that KEAP1 expression was elevated in most cancers in both non-paired samples and paired samples except in KICH compared with paired normal tissues (Figures 1B,C). These results suggest that KEAP1 expression may play an important role in the development of liver cancer. Therefore, we further analyzed the correlation between KEAP1 expression and LIHC prognosis. Kaplan–Meier survival analysis revealed that although KEAP1 expression was not a significant predictor of disease-specific survival and progress free interval, KEAP1 expression is associated with overall survival (hazard ratio [HR] = 1.44) in LIHC patients (Figure 1D). Furthermore, we analyzed the relationship between KEAP1 expression and overall survival of LIHC patients during each period. It was found that KEAP1 expression was significantly correlated with the survival time of LIHC patients with different histologic grade (HR = 1.49), pathologic stage (HR = 1.47), and T stage (HR = 1.47) (Figure 1E). LIHC patients with high KEAP1 expression had lower overall survival than those with low KEAP1 expression. At the same time, LIHC patients with dead outcome had higher KEAP1 expression than those with alive outcome (Figure 1F). Besides, KEAP1 expression showed great diagnostic value (AUC, 0.912) with significant high sensitivity of 0.912 and specificity of 0.853 to identify LIHC tissues from normal tissues (Figure 1G). These results suggest that KEAP1 expression is significantly elevated and is closely related to prognosis in LIHC patients. Detailed data are presented in Supplementary Table S2: S1–S6.

### 3.2 Associations between KEAP1 expression and clinicopathologic characteristics in LIHC

We extracted and analyzed the demographic and clinical data of LIHC patients with low- (187) and high-KEAP1 expression (187) from the TCGA (Table 2). We further used single gene logistics regression analysis to determine the correlation between KEAP1 expression and clinicopathological characteristics in LIHC patients (Table 3). It was found that KEAP1 expression was positively correlated with pathological stage of LIHC patients (Odds Ratio [OR] = 1.583 for Stage II&Stage III&Stage IV vs. Stage I). To better understand the relevance and mechanisms of KEAP1 expression in LIHC, we investigated the relationship between KEAP1 expression and the clinical characteristics of LIHC patients by univariate Cox regression analysis (Table 4). It was found that high KEAP1 expression correlated with poor survival of LIHC patients. Other clinical characteristics that correlated with poor survival included high T stage, high M stage, high pathologic stage, and with tumor status. To further explore factors associated with survival, multivariate Cox regression analysis was performed, which revealed that tumor status (HR = 1.819) was an independent risk factor for overall survival (Table 4). These results suggest that KEAP1 expression may be correlated with overall survival in LIHC patients and deserves further study.

**Table 2:** Demographic and clinical characteristics of LIHC patients with low- and high-expression of KEAP1 in TCGA.

Characteristic	Low expression of KEAP1	High expression of KEAP1	*p*
*n*	187	187	
Age, *n* (%)			0.715
<=60	91 (24.4%)	86 (23.1%)	
>60	96 (25.7%)	100 (26.8%)	
Gender, *n* (%)			1.000
Female	61 (16.3%)	60 (16%)	
Male	126 (33.7%)	127 (34%)	
Race, *n* (%)			0.273
Asian	77 (21.3%)	83 (22.9%)	
Black or African American	6 (1.7%)	11 (3%)	
White	99 (27.3%)	86 (23.8%)	
T stage, *n* (%)			0.182
T1	101 (27.2%)	82 (22.1%)	
T2	44 (11.9%)	51 (13.7%)	
T3	37 (10%)	43 (11.6%)	
T4	4 (1.1%)	9 (2.4%)	
N stage, *n* (%)			1.000
N0	124 (48.1%)	130 (50.4%)	
N1	2 (0.8%)	2 (0.8%)	
M stage, *n* (%)			1.000
M0	137 (50.4%)	131 (48.2%)	
M1	2 (0.7%)	2 (0.7%)	
Pathologic stage, *n* (%)			0.149
Stage I	97 (27.7%)	76 (21.7%)	
Stage II	40 (11.4%)	47 (13.4%)	
Stage III	36 (10.3%)	49 (14%)	
Stage IV	3 (0.9%)	2 (0.6%)	
Histologic grade, *n* (%)			0.419
G1	31 (8.4%)	24 (6.5%)	
G2	93 (25.2%)	85 (23%)	
G3	55 (14.9%)	69 (18.7%)	
G4	6 (1.6%)	6 (1.6%)	
AFP (ng/mL), *n* (%)			0.895
<=400	110 (39.3%)	105 (37.5%)	
>400	32 (11.4%)	33 (11.8%)	
Albumin (g/dl), *n* (%)			0.656
<3.5	38 (12.7%)	31 (10.3%)	
>=3.5	118 (39.3%)	113 (37.7%)	
Prothrombin time, *n* (%)			0.455
<=4	103 (34.7%)	105 (35.4%)	
>4	49 (16.5%)	40 (13.5%)	
Child-Pugh grade, *n* (%)			0.495
A	114 (47.3%)	105 (43.6%)	
B	9 (3.7%)	12 (5%)	
C	1 (0.4%)	0 (0%)	

Cases with insufficient or missing data were removed from data processing.

**Table 3:** Logistic regression analysis of association between clinicopathological characteristics and KEAP1 expression in LIHC patients.

Characteristics	Total (*N*)	Odds ratio (OR)	*p* value
T stage (T2&T3&T4 vs. T1)	371	1.493 (0.992–2.251)	0.055
N stage (N1 vs. N0)	258	0.954 (0.113–8.049)	0.963
M stage (M1 vs. M0)	272	1.046 (0.124–8.819)	0.965
Pathologic stage (Stage II & Stage III & Stage IV vs. Stage I)	350	1.583 (1.040–2.419)	0.033
Tumor status (With tumor vs. Tumor free)	355	1.284 (0.843–1.958)	0.245
Age (>60 vs. <=60)	373	1.102 (0.734–1.656)	0.639
Gender (Male vs. Female)	374	1.025 (0.664–1.582)	0.912
Residual tumor (R1&R2 vs. R0)	345	1.321 (0.508–3.540)	0.568
Histologic grade (G2&G3&G4 vs. G1)	369	1.342 (0.756–2.408)	0.318
AFP(ng/mL) (>400 vs. <=400)	280	1.080 (0.620–1.886)	0.785
Child-Pugh grade (B&C vs. A)	241	1.303 (0.540–3.208)	0.556

Cases with insufficient or missing data were removed from data processing.

**Table 4:** Association of clinicopathological characteristics with overall survival using univariate or multivariate cox regression analysis.

Characteristics	Total (*N*)	Univariate analysis		Multivariate analysis	
		Hazard ratio (95% CI)	*p* value	Hazard ratio (95% CI)	*p* value
Age	373				
<=60	177	Reference			
>60	196	1.205 (0.850–1.708)	0.295		
Gender	373				
Female	121	Reference			
Male	252	0.793 (0.557–1.130)	0.200		
T stage	370				
T1	183	Reference			
T2	94	1.431 (0.902–2.268)	0.128	0.000 (0.000-Inf)	0.995
T3	80	2.674 (1.761–4.060)	<0.001	0.952 (0.127–7.130)	0.962
T4	13	5.386 (2.690–10.784)	<0.001	1.793 (0.207–15.561)	0.596
N stage	258				
N0	254	Reference			
N1	4	2.029 (0.497–8.281)	0.324		
M stage	272				
M0	268	Reference			
M1	4	4.077 (1.281–12.973)	0.017	2.186 (0.159–30.109)	0.559
AFP(ng/mL)	279				
<=400	215	Reference			
>400	64	1.075 (0.658–1.759)	0.772		
Pathologic stage	349				
Stage I	173	Reference			
Stage II	86	1.417 (0.868–2.312)	0.164	4329290.072 (0.000-Inf)	0.994
Stage III	85	2.734 (1.792–4.172)	<0.001	2.933 (0.385–22.368)	0.299
Stage IV	5	5.597 (1.726–18.148)	0.004		
Tumor status	354				
Tumor free	202	Reference			
With tumor	152	2.317 (1.590–3.376)	<0.001	1.819 (1.136–2.914)	0.013
Histologic grade	368				
G1	55	Reference			
G2	178	1.162 (0.686–1.969)	0.576		
G3	123	1.185 (0.683–2.057)	0.545		
G4	12	1.681 (0.621–4.549)	0.307		
Residual tumor	344				
R0	326	Reference			
R1&R2	18	1.604 (0.812–3.169)	0.174		
Child-Pugh grade	240				
A	218	Reference			
B&C	22	1.643 (0.811–3.330)	0.168		
KEAP1	373				
Low	187	Reference			
High	186	1.436 (1.015–2.031)	0.041	1.262 (0.805–1.980)	0.310

Cases with insufficient or missing data were removed from data processing.

### 3.3 KEAP1 related functional annotation and signaling pathways based on GO and GSEA

**Figure 2:**
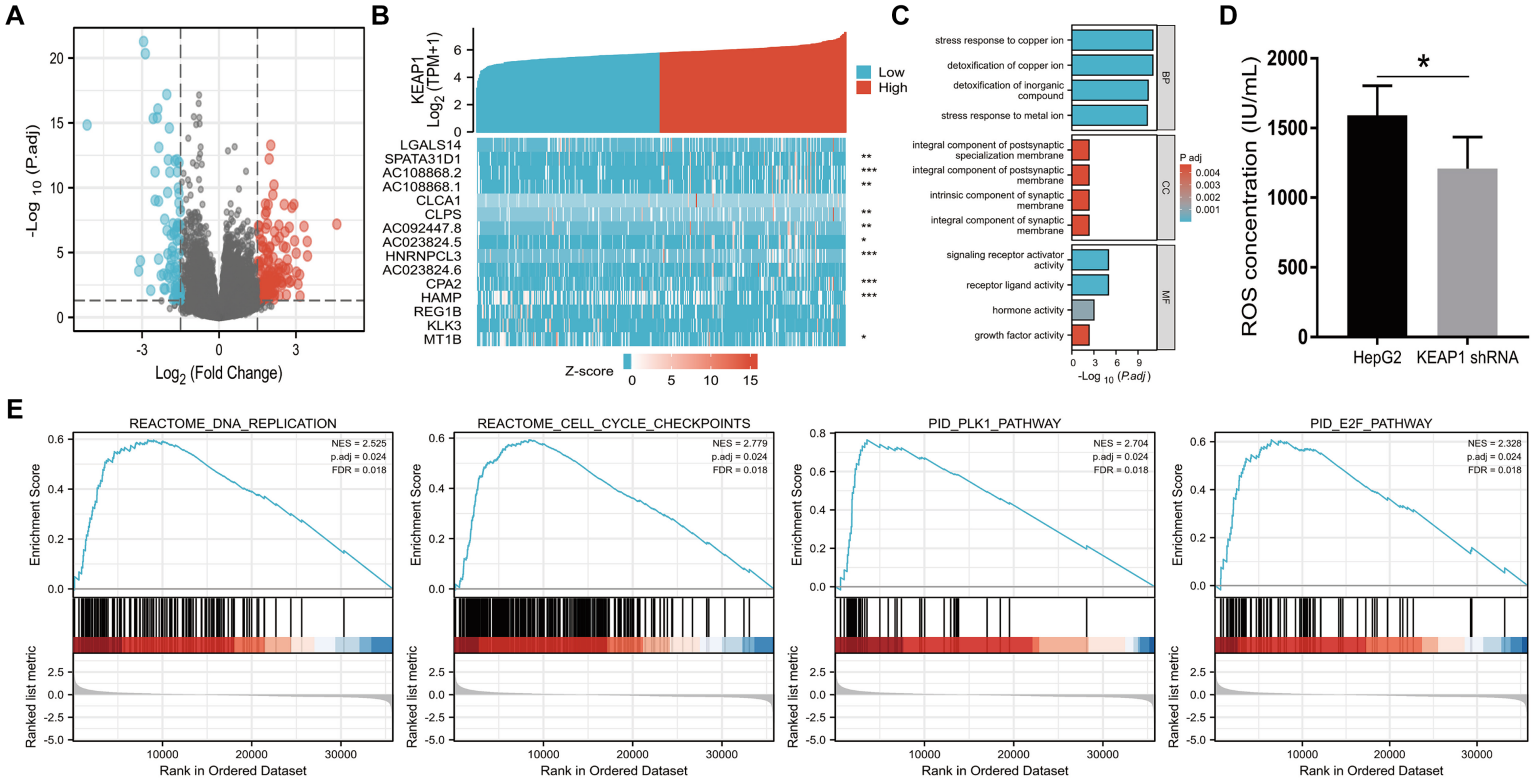
KEAP1 related functional annotation and signaling pathways based on GO and GSEA. **(A)** 153 upregulated and 78 downregulated genes between LIHC patients with high- and low-KEAP1 expression based on the criteria of logFC >1.5 and adjusted *p*-value <0.05. **(B)** Absolute logFC value of the top 15 DEGs between LIHC patients with high- and low-KEAP1 expression. **(C)** GO enrichment analysis for DEGs identified between LIHC patients with high- and low-KEAP1 expression groups. **(D)** ROS levels of hepG2 cells was significantly reduced after inhibiting Keap1 expression. KEAP1 shRNA was used to reduce KEAP1 expression in HepG2 cells. **(E)** GSEA for DEGs identified between LIHC patients with high- and low-KEAP1 expression groups. GO, gene ontology; DEGs, differentially expressed genes; ROS, reactive oxygen species.

Results above suggest that KEAP1 expression may play an important role in the prognosis of LIHC patients and is correlated with the overall survival of LIHC patients. Therefore, we conducted relevant studies focusing on KEAP1 expression in LIHC patients, and further extracted and analyzed 56,493 genes from TCGA between patients with high- and low-KEAP1 expression using DESeq2 [1.26.0 version] (14) in R (Supplementary Table S3: S1). There were 231 DEGs between the two groups based on the criteria of logFC >1.5 and p.adj < 0.05, covering 153 upregulated and 78 downregulated genes (Figure 2A) (Supplementary Table S3: S2). Then, DEGs in the HTSeq-Counts were further analyzed by ggplot2 [3.3.3 version] in R. Absolute logFC value of the top 15 DEGs between the two groups were illustrated by heatmaps (Figure 2B). To better understand the functional implications of the 231 DEGs, GO enrichment analysis was performed using the ClusterProfile package (11). The significance cut-off value of GO enrichment analysis was set as p.adj < 0.05, and 30 enriched terms were identified in the GO “biological process” category, including “stress response to copper ion,” “detoxification of copper ion,” “stress response to metal ion,” and “detoxification of inorganic compound”; 13 enriched terms were identified in the GO “cellular component” category, including “integral component of postsynaptic (synaptic) membrane” and “intrinsic component of synaptic membrane”; and 5 enriched terms were identified in the GO “molecular function” category, including “signaling receptor activator activity,” “receptor ligand activity,” “hormone activity,” and “growth factor activity” (Figure 2C) (Supplementary Table S3: S3). These results suggest that abnormally expressed KEAP1 is associated with the stress response and detoxification of copper ion, synaptic composition, and signaling conduction. Besides, ROS levels in hepatocellular carcinoma cells were detected to assist in determining the oxidative stress role of Keap1 in LIHC. The results showed that under the same stimulation condition, the ROS level of hepatocellular carcinoma cells was significantly reduced after inhibiting Keap1 expression, suggesting that the high expression of Keap1 may promote oxidative stress of hepatocellular carcinoma cells (Figure 2D) (Supplementary Table S3: S4).

To identify key pathways related to KEAP1 expression in LIHC, GSEA was used between the high- and low-KEAP1 expression groups. The results showed that KEAP1 related DEGs mainly enriched in cell development and signal transduction, such as “REACTOME_DNA_REPLICATION” [normalized enrichment score (NES) = 2.525, p.adj = 0.024, FDR = 0.018], “REACTOME_CELL_CYCLE_CHECKPOINTS” [NES = 2.779, p.adj = 0.024, FDR = 0.018], “PID_PLK1_PATHWAY” [NES = 2.704, p.adj = 0.024, FDR = 0.018], “PID_E2F_PATHWAY” [NES = 2.328, p.adj = 0.024, FDR = 0.018] (Figure 2E) (Supplementary Table S3: S5).

### 3.4 Correlation between KEAP1 expression and immune infiltration in LIHC

**Figure 3:**
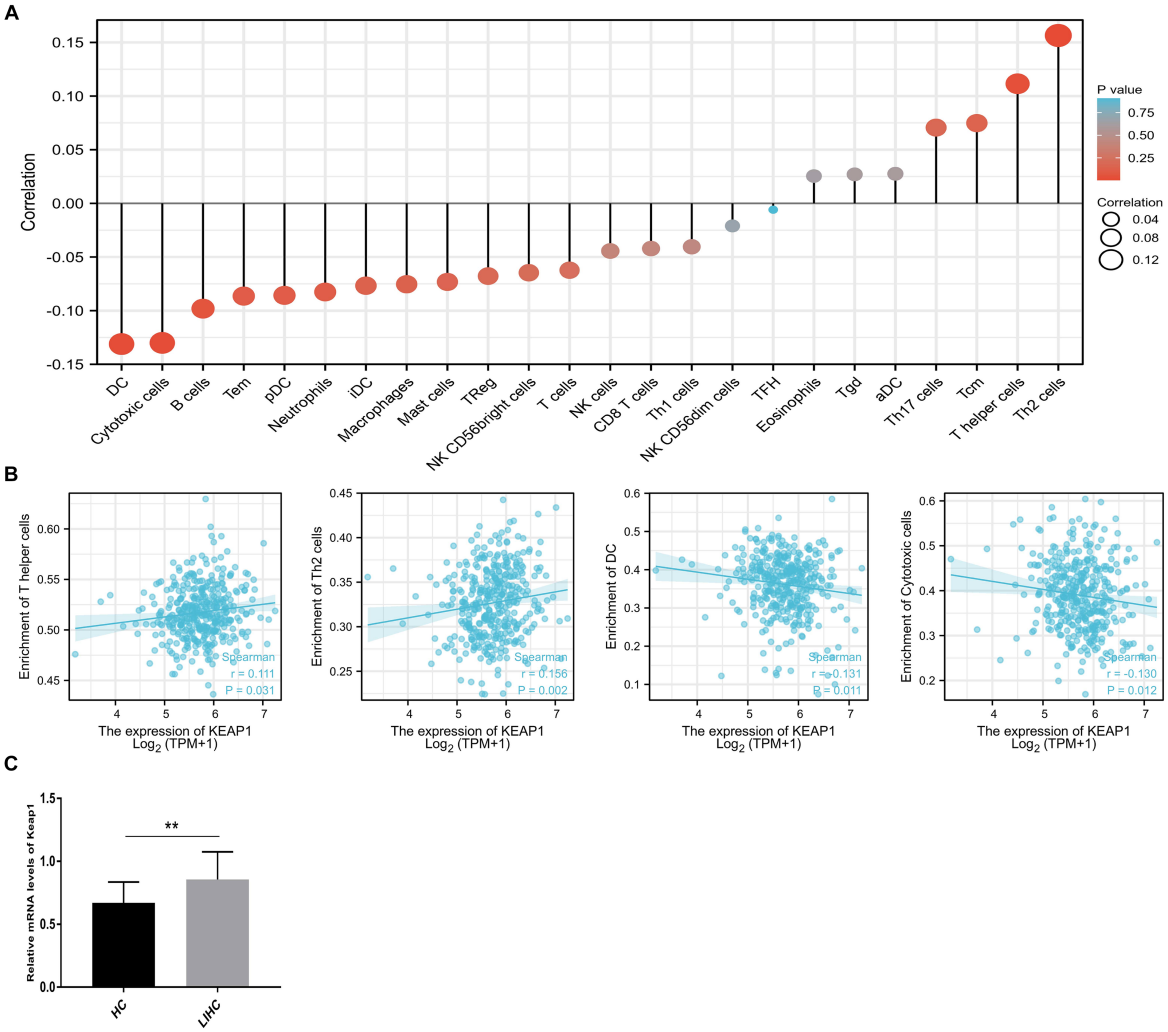
Correlation analysis of KEAP1 expression and immune infiltration in LIHC. **(A)** Single-sample GSEA was used to analyze the relationship between KEAP1 expression and immune cell enrichment in LIHC. **(B)** KEAP1 expression was positively correlated with the infiltration level of T helper cells and Th2 cells, while negatively correlated with DC and cytotoxic cells. **(C)** KEAP1 mRNA level of circulating lymphocytes in LIHC patients was significantly higher than that in HCs.

Considering that KEAP1 may participate in cell development and signal transduction from the results of GO and GSEA, and the important role of cellular immunity in cancer progression. We further applied ssGSEA to analyze the relationship between KEAP1 expression and immune cell enrichment in LIHC (Figure 3A). We found that KEAP1 expression was positively correlated with the infiltration level of T helper cells (*r* = 0.111, *p* = 0.031) and Th2 cells (*r* = 0.156, *p* = 0.002), while negatively correlated with the infiltration level of DC (*r* = −0.131, *p* = 0.011), and cytotoxic cells (*r* = −0.130, *p* = 0.012) (Figure 3B). Besides, we detected KEAP1 mRNA levels of circulating lymphocytes, and found that KEAP1 mRNA levels of circulating lymphocytes in LIHC patients was significantly higher than that in HCs (Figure 3C). Detailed data are presented in Supplementary Table S4.

### 3.5 Functional changes of KEAP1 expression related immune infiltration cells in LIHC

**Figure 4:**
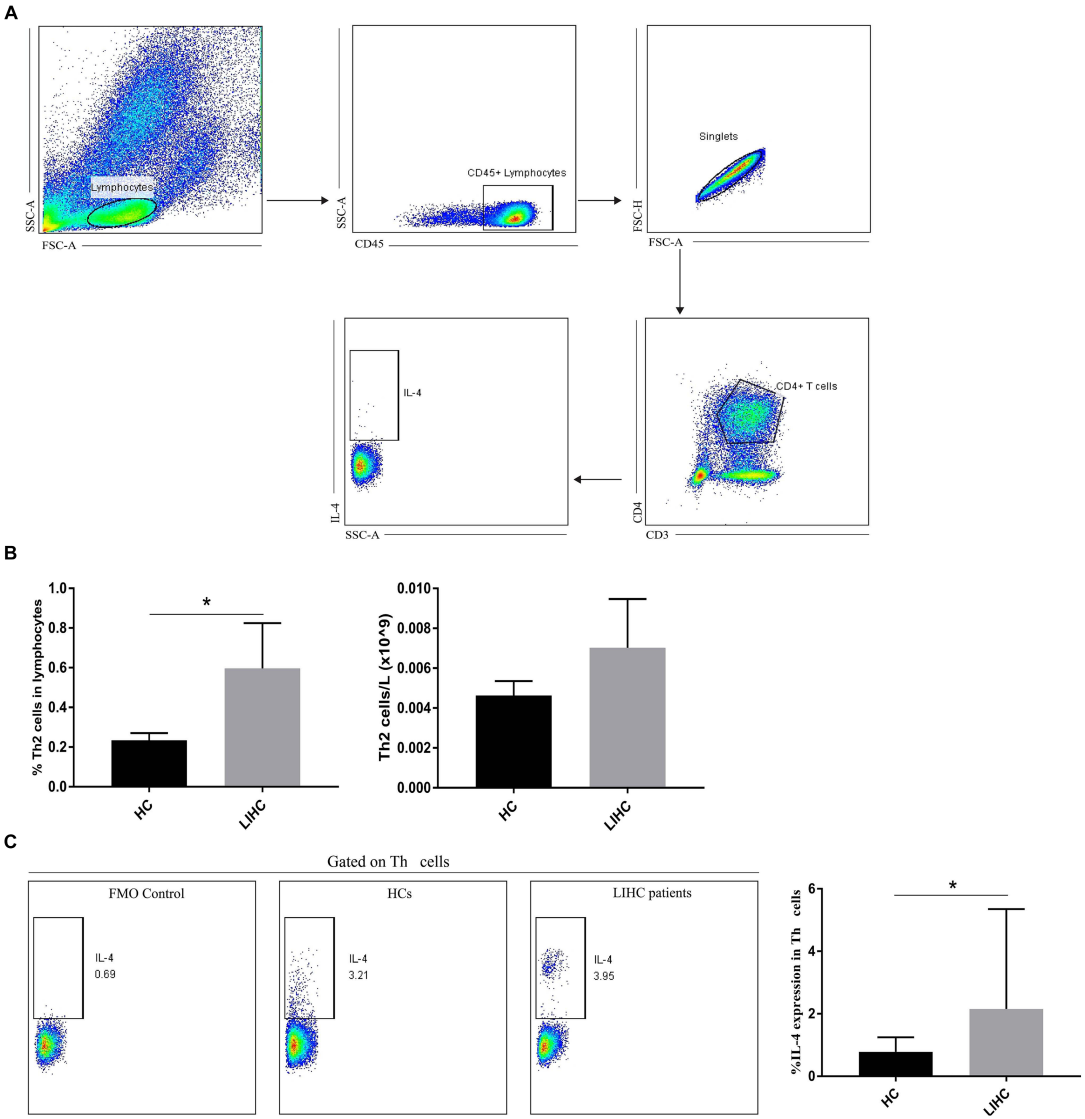
Changes of Th2 cells in LIHC patients. **(A)** Gate strategy of IL-4 expression in Th2 cells in flow cytometry. **(B)** The frequency of Th2 cells in LIHC patients was significantly higher than that in HCs, but the absolute number of Th2 cells was not significant different between the two groups. **(C)** The expression of IL-4 in Th2 cells of LIHC patients was significantly higher than that of HCs. FMO (fluorescence minus one) staining was used to distinguish positive from negative thereby helping circle the gate more accurately. HCs, healthy controls.

**Figure 5:**
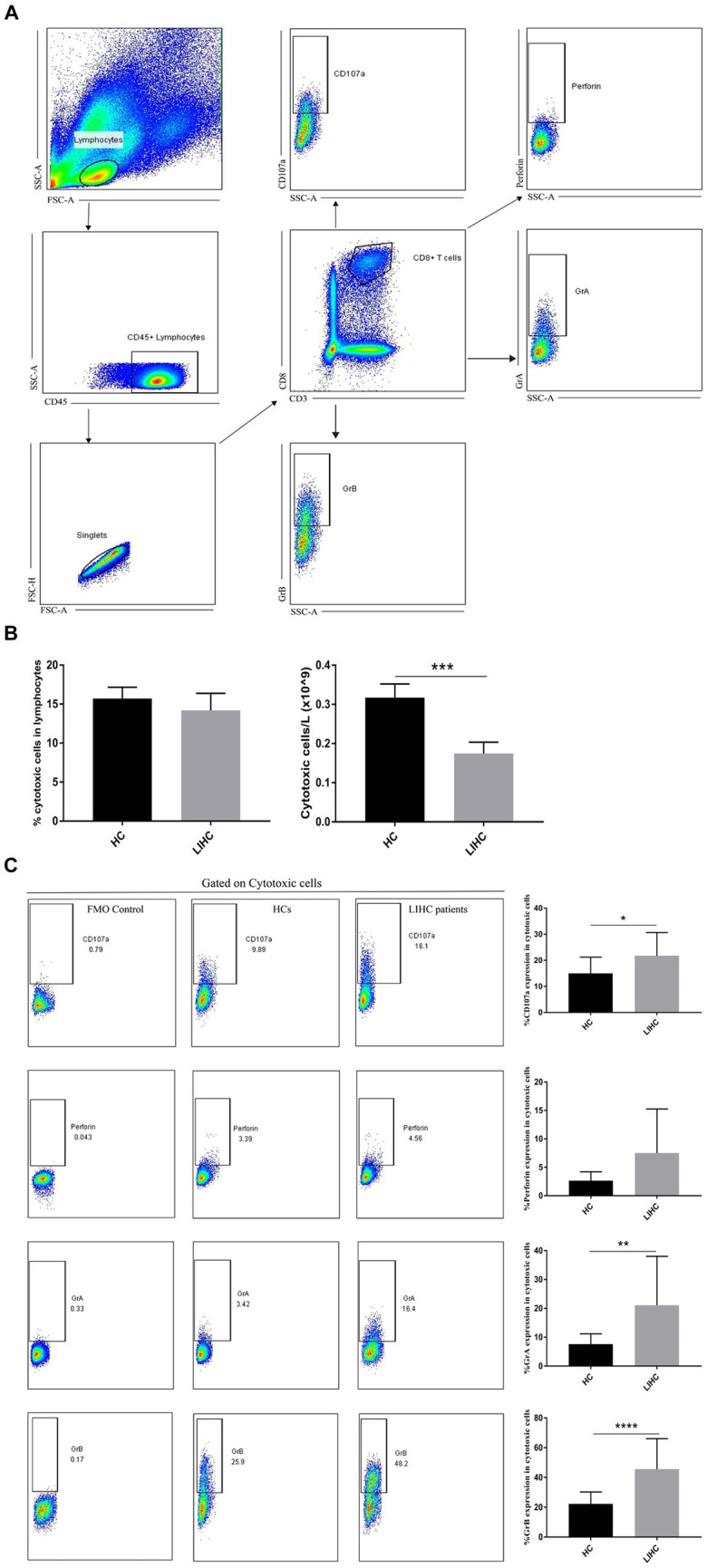
Changes of cytotoxic cells in LIHC patients. **(A)** Gate strategy of cytotoxic cell-related cytokine expression in flow cytometry. **(B)** The frequency of cytotoxic cells was not significant different between the two groups, the absolute number of cytotoxic cells in LIHC patients was significantly lower than that in HCs. **(C)** Compared to HCs, the expression of CD107a, GrA and GrB in cytotoxic cells of LIHC patients was significantly increased, while the expression of perforin was not significantly changed. GrA, granzyme A; GrB, granzyme B.

The correlation analysis suggest that KEAP1 expression is closely related to immune infiltration in LIHC, so it is particularly necessary to analyze the function of relevant immune cells to assist in judging the immune status of LIHC patients. We selected two broad cell populations (Th2 cells and cytotoxic cells) that are positively and negatively correlated with KEAP1 expression in LIHC patients for functional analysis. Th2 cells mainly mediate immune regulation by secreting IL-4. Therefore, the expression level of IL-4 was analyzed. It was found that the frequency of Th2 cells in LIHC patients was significantly higher than that in HCs, but the absolute number of Th2 cells was not significant different between the two groups (Figures 4A,B). Besides, the expression of IL-4 in Th2 cells of LIHC patients was significantly higher than that of HCs (Figure 4C). Cytotoxic cells secrete perforin and granzyme to mediate the killing function through degranulation. It was found that although the frequency of cytotoxic cells was not significant different between the two groups, the absolute number of cytotoxic cells in LIHC patients was significantly lower than that in HCs (Figures 5A,B). Besides, the expression level of CD107a, GrA and GrB in cytotoxic cells of LIHC patients were significantly higher than that of HCs, but there was no difference in the expression level of perforin between the two groups (Figure 5C). These results suggest that Th2 and cytotoxic cells in LIHC patients may be in an immunoactivated state, and their immune activity is significantly enhanced. Detailed data are presented in Supplementary Table S5.

### 3.6 KEAP1 expression is closely related to liver function in LIHC

**Figure 6:**
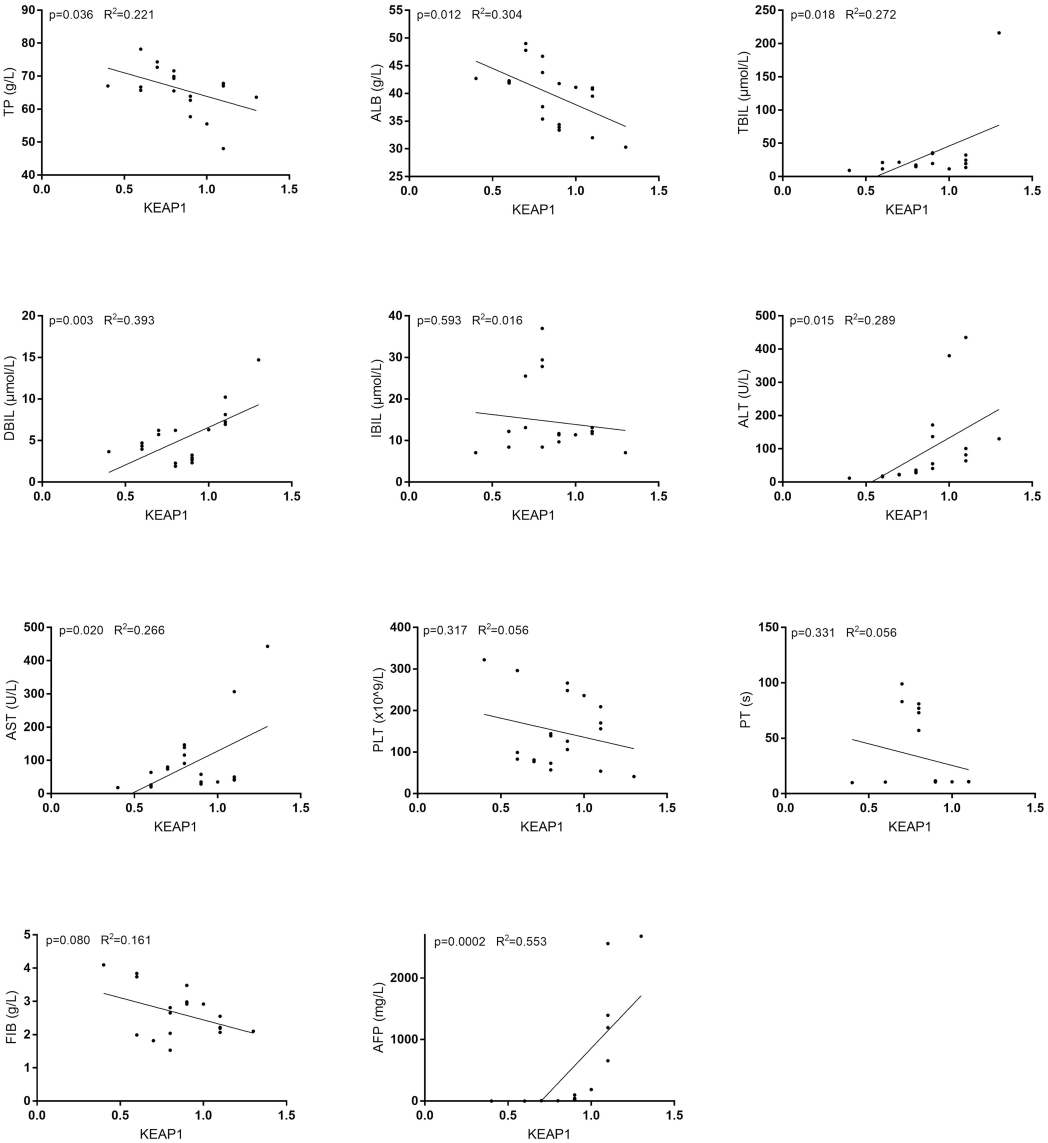
KEAP1 expression is closely related to liver function in LIHC. KEAP1 expression was significantly negatively correlated with the level of TP and ALB, and positively correlated with TBIL, DBIL, ALT, AST and AFP, but not correlated with IBIL, PLT, PT, and FIB. TP, total protein; ALB, albumin; TBIL, total bilirubin; DBIL, direct bilirubin; IBIL, indirect bilirubin; PLT, platelet; PT, prothrombin time; FIB, fibrinogen.

Results above suggest that KEAP1 may play an important role in the development of LIHC. Therefore, we further investigated the relationship between KEAP1 expression and liver function. Correlation analysis revealed that KEAP1 expression was significantly negatively correlated with the level of total protein (TP) and albumin (ALB), and positively correlated with total bilirubin (TBIL), direct bilirubin (DBIL), ALT, AST, and AFP, but not correlated with indirect bilirubin (IBIL), platelet (PLT), prothrombin time (PT) and fibrinogen (FIB) (Figure 6) (Supplementary Tables S1, S4). These results suggest that KEAP1 expression is closely related to liver function in LIHC patients, and elevated KEAP1 expression may promote liver injury.

## 4 Discussion

Considering the difficulty of treatment and poor prognosis of LIHC, it is extremely necessary to provide timely and effective targets for clinical diagnosis and treatment of LIHC, so as to improve the survival time of patients. KEAP1 has been found to be abnormally elevated in many cancers, such as HNSC, LUSC (15, 16). In this study, we found that KEAP1 was significantly overexpressed in LIHC patients, and found that KEAP1 has a good diagnostic value for LIHC (AUC = 0.912) with high sensitivity (0.912) and specificity (0.853). Therefore, it is necessary to further explore the clinical value of KEAP1 in LIHC. Kaplan–Meier survival analysis and logistics regression analysis revealed that KEAP1 expression was correlated with overall survival of LIHC patients, especially in patients with advanced histologic grade, pathologic stage and T stage. These results suggest that KEAP1 expression is closely related to the diagnosis and prognosis of LIHC. Therefore, we followed up with relevant studies on KEAP1 expression in LIHC. We identified 231 DEGs between LIHC patients with high- and low-KEAP1 expression. GO analysis revealed that these DEGs were mainly involved in stress response and detoxification of inorganic substances, synaptic composition, and signaling conduction. Inorganic is an indispensable part of many key biological pathways, and its abnormal distribution may lead to abnormal cell function and thus biological dysfunction. Rodrigues JFV and colleagues reported that human cells treated with silver nanoparticles upregulated mineral absorption, ferroptosis, protein processing in the endoplasmic reticulum, and mitogen-activated protein kinase signaling pathway expression. At the same time, inorganic compounds and oxidative stress response genes were shared, triggering apoptosis (17). In LIHC patients, KEAP1 expression was closely related to stress response and detoxification of inorganic compounds. Therefore, the mechanism and related pathways of KEAP1 expression regulating the inorganic distribution of LIHC are worth further investigation. The formation of intact synapses is an important part of signal transmission, usually releasing neurotransmitters from the presynaptic membrane to the postsynaptic membrane to transmit excitatory or inhibitory signals (18). Synaptic changes caused by severe stress or trauma can lead to abnormal signal transmission, and the release of a large number of neurotransmitters in the short term may cause irreversible damage to related brain areas, such as the amygdala, and induce mood disorders and mental disorders (19). Differential genes related to KEAP1 expression in LIHC patients are associated with synaptic composition, but their role in regulating patients’ emotions and related mechanisms remain unclear, which may be worth exploring. In addition, signaling is also an important part for cells to play different biological functions. The expression of different molecules in cells can be regulated by direct contact between cells or sensing changes in the composition of intercellular fluid to play different functions (20). Therefore, whether the signaling related differential gene involved in KEAP1 expression in LIHC patients acts on hepatocytes and related peripheral cells, as well as the effect of its chain reaction on the occurrence and development of LIHC and the related mechanism are questions worth exploring.

In addition, we used GSEA to further analyze key pathways involved in KEAP1 expression related genes in LIHC and found that they are mainly focused on cell development and signal transduction. These results suggest that KEAP1 has a good correlation with LIHC, and the differential genes related to its expression are involved in stress response, synaptic formation, signal transduction, cell development, etc., and are inextricably related to the normal play of cell function. Therefore, it is necessary to further study the relationship between KEAP1 expression and oxidative stress and cell function. We examined the role of KEAP1 in regulating oxidative stress in hepatocellular carcinoma cells, and found that inhibiting KEAP1 expression could significantly reduce ROS levels in hepatocellular carcinoma cells, suggesting that high KEAP1 expression may promote cell dysfunction. Tu et al. systematically introduced the role of KEAP1 in coordination with NRF2 and ARE in mediating and inhibiting inflammation and oxidative stress in various chronic diseases (21), suggesting that KEAP1 may promote inflammation and oxidative stress by inhibiting downstream molecules NRF2 and ARE. Besides, relevant studies have explored the KEAP1-targeting molecule PGAM5 based on the Keap1/Nrf2/ARE pathway, and found that enhancing the expression of PGAM5 can significantly inhibit the expression of KEAP1, thus inhibiting the production of ROS, which is of great value for improving disease progression (22, 23). These studies suggest that the high expression of KEAP1 in LICH tissue may promote the progression of inflammation and oxidative stress in patients, and may improve disease progression in LIHC patients by discovering molecules targeting KEAP1.

Immune cells are the main body of immune system to perform immune function. They recognize foreign antigens through specific receptors, directly or indirectly eliminate foreign pathogens, mutated tumor cells and damaged senescent cells, and regulate intercellular immunity by secreting related cytokines to transmit specific information, mediating the normal functions of tissues and the body (16). Therefore, the dysfunction of immune cells may seriously affect the body’s health. This study found that KEAP1 expression is closely related to immune cell infiltration, which indirectly proves that keap1 expression may affect the occurrence and development of LIHC. It was found that KEAP1 expression was positively correlated with the infiltration level of T helper cells and Th2 cells, while negatively correlated with the infiltration level of DC and cytotoxic cells, these data can help us to more conveniently and quickly screen out the cell populations that have a greater likelihood of affecting the development of tumors, so as to further perform functional analysis of these cell populations to assist in judging the immune status of LIHC patients. Th cells recognize antigen fragments presented by MHC molecules of antigen-presenting cells mainly through specific receptors on the cell surface, stimulate intracellular cascade reactions, induce secretion of related cytokines, and then regulate the function of target cells and play a role in immune regulation (24). DC can efficiently take up, process and present antigens. Immature DC has strong migration ability, and mature DC can effectively activate the initial T cells, which is the central link to initiate, regulate and maintain the immune response. DC, as the most functional antigen-presenting cells discovered so far, can induce the generation of specific cytotoxic T lymphocytes (25). Cytotoxic T lymphocytes are extremely important members of anti-virus and anti-tumor immunity, they kill target cells by directly contacting or secreting perforin and granzyme, which is an indispensable part of immune defense and immune surveillance (26). In this study, two widespread cell groups (Th2 cells and Cytotoxic cells), which are positively and negatively correlated with KEAP1 expression in LIHC, were selected for functional analysis to assist in judging the immune status of LIHC patients. It was found that the expression level of IL-4 in Th2 cells of LIHC patients was significantly increased, suggesting that its immune regulatory function might be enhanced. In addition, the expression of CD107a in cytotoxic cells of LIHC patients was significantly enhanced, suggesting increased degranulation ability. Perforin and granzyme are the main molecules of cytotoxic cells mediating killing function and are secreted by degranulation. Therefore, we further examined the expression level of perforin and granzyme in cytotoxic cells of LIHC patients. The results showed that as compared with HCs, although there was no significant change in the expression of perforin in cytotoxic cells of LIHC patients, the expression levels of granzyme A and granzyme B were significantly increased, suggesting that the immune activity of cytotoxic cells in LIHC patients may be enhanced. These results suggest that there may be significant changes in immune cell function in LIHC patients, but the effects of these changes on body immunity and liver immunity, as well as the effects on liver injury and repair still need to be further explored. Besides, we have not further studied the effect of abnormal KEAP1 expression on immune cell function, which is our shortcoming and one of the research directions to be carried out in the future. In addition, our study found that KEAP1 expression was associated with liver function related indicators, and its increase was positively correlated with the increase of aminotransferase and bilirubin, while negatively correlated with the level of TP and ALB, suggesting that the increase of KEAP1 expression may mediate liver injury. Wang et al. found that TNF-α can promote ECV304 cell injury, and this injury may be inhibited by regulating KEAP1-Nrf2 signaling pathway, suggesting that KEAP1 plays a role in cell injury through related pathways (27). However, the mechanism of how KEAP1 mediates liver injury in LIHC patients is still unclear and deserves further study.

In conclusion, this study revealed that highly expressed KEAP1 is closely related to the diagnosis, prognosis, immune infiltration, and liver function of LIHC, which might promote the progression of LIHC through regulating cell development, signal transduction, and abnormal immune responses. The current study partially revealed the role of KEAP1 in LIHC and may have positive implications for enriching the diagnosis, prognosis and treatment of LIHC.

## Data availability statement

The datasets presented in this study can be found in online repositories. The names of the repository/repositories and accession number(s) can be found in the article/Supplementary material.

## Ethics statement

The studies involving humans were approved by the ethics committee of the First Affiliated Hospital of Anhui Medical University. The studies were conducted in accordance with the local legislation and institutional requirements. The participants provided their written informed consent to participate in this study.

## Author contributions

XW: Data curation, Formal analysis, Writing – original draft, Writing – review & editing. YT: Investigation, Software, Writing – original draft. MZ: Conceptualization, Formal analysis, Project administration, Writing – review & editing. YX: Conceptualization, Formal analysis, Project administration, Writing – review & editing. ZW: Conceptualization, Formal analysis, Project administration, Writing – review & editing.

## Footnotes

^1^ https://portal.gdc.cancer.gov

^2^ http://www.sthda.com/english/wiki/r-packages

^3^ https://www.gsea-msigdb.org/gsea/index.jsp

^4^ https://www.gsea-msigdb.org/gsea/msigdb/
